# Supplementary material for: Ultraviolet photolysis of H2S and its implications for SH radical production in the interstellar medium
Source: Nat Commun. 2020 Mar 24;11:1547. doi: 10.1038/s41467-020-15343-4 (PMC7093389; doi:10.1038/s41467-020-15343-4)
Supplement: Supplementary file 1 — Supplementary Information [file 41467_2020_15343_MOESM1_ESM.pdf]

Supplementary Information

Ultraviolet Photolysis of H_2S and its Implications for SH Radical Production in the Interstellar Medium

Zhou et al

Supplementary Table 1. Thermochemical threshold energies for fragmentation processes relevant to the present study.

**Dissociation products**	**Threshold energy/cm^{-1}**	**Reference**
H + SH($X^2\Pi_{3/2}$, v= 0, N= 1)	31451 ± 4	1
H + SH($A^2\Sigma$, v' = 0, N' = 0)	62284 ± 4	1,2
H + H + S($^3P_2$)	60696 ± 25	1,3
H + H + S($^1D_2$)	69935 ± 25	1,3,4
H_2($X^1\Sigma_g^+$, v″= 0, j''= 0) + S($^1D_2$)	33817 ± 25	1,3,4,5

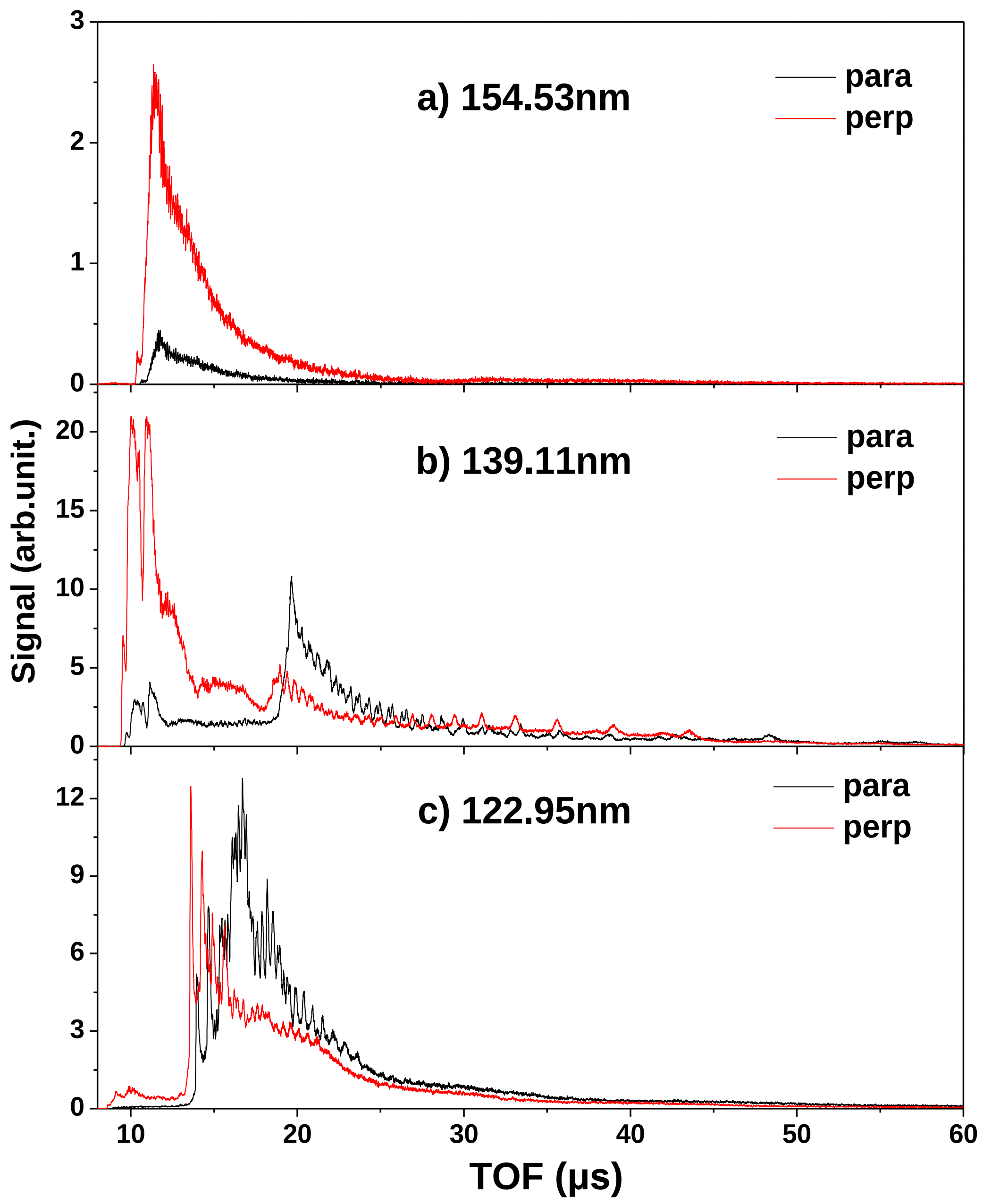

Supplementary Figure 1. Time of flight spectra of the H atom products from photodissociation of H_2S at (a) 154.53 nm, (b) 139.11 nm and (c) 122.95 nm with the detection axis parallel (black) and perpendicular (red) to the photolysis laser polarization.

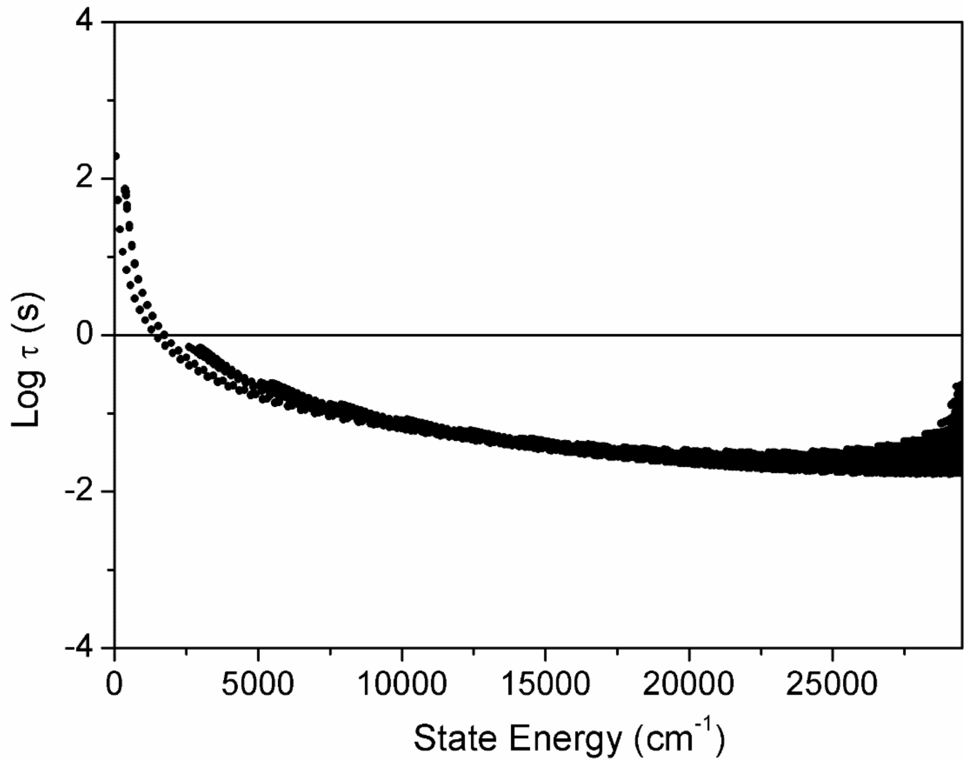

Supplementary Figure 2. Plot showing the internal energy dependence of the radiative lifetime of SH(X) radicals (from ref. 6).

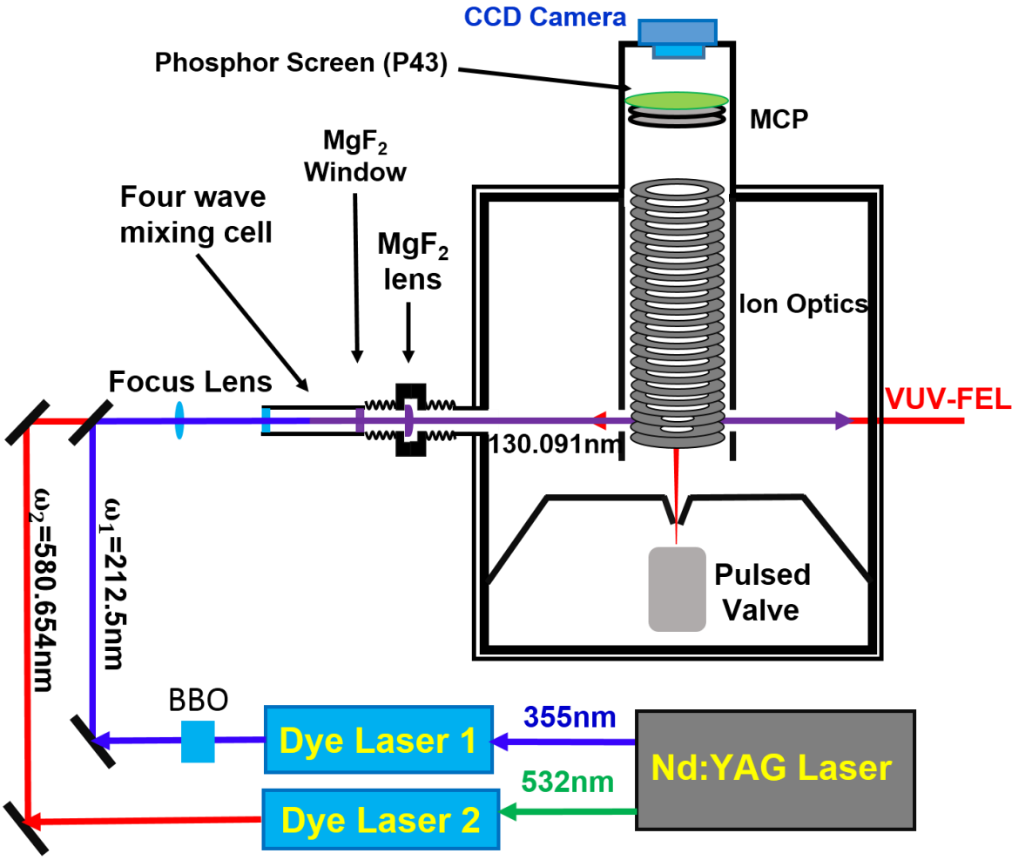

Supplementary Figure 3. Schematic of the two-colour VUV FEL-VMI experimental setup.

Supplementary Note 1

Radiative lifetimes of SH(X) rovibrational levels. Radiative lifetimes, τ_{rad}, for individual SH(X, *v''*, *N''*) levels are available directly from a recent addition to the ExoMol database by Gorman *et al.*[6] and are plotted as a function of internal energy in Supplementary Figure 2. The τ_{rad} values for the higher *v''*, *N''* states populated when photodissociating H_2S at *e.g.* λ = 154.53 or 139.11 nm are typically ~0.1 sec, largely determined by the $\Delta v = -1$ transition probabilities (augmented by larger *v''* changing transitions when available). The τ_{rad} values increase at low internal energies, as radiative decay is restricted to just v''=1$\rightarrow$$v''$=0 transitions and, ultimately, to pure rotational transitions within the v''=0 manifold of levels.

Supplementary note 2

Experimental Methods. The vacuum ultraviolet free electron laser (VUV-FEL) and the H-atom Rydberg atom TOF experiment have been described previously [7] and details are not repeated here. However, the procedures for undertaking velocity map imaging (VMI) data using the FEL output has not been described previously. The experiment involves a molecular beam, photolysis and probe lasers and the detection system and is summarized in Supplementary Figure 3.

The pulsed supersonic beam was generated by expanding a mixture of 1% H_2S and Ar at a backing pressure of 1 bar into the source chamber where it was skimmed before entering the ion optics assembly (IOA, 23-plate ion optics [8]) mounted in the differentially pumped reaction chamber. The beam passed through a 2 mm hole in the first electrode and propagates along the centre axis of the IOA towards the centre of the front face of the detector. The molecular beam was intersected at right angles by the counter-propagating photolysis and probe laser beams between the second and the third plates of the IOA. The photolysis photons were provided by the FEL, operating at 10 Hz with $\boldsymbol{\varepsilon}_{\mathbf{pump}}$ fixed in the horizontal plane and thus parallel to the front face of the detector. The $S(^1D_2)$ photoproducts were probed by one photon excitation at λ = 130.091 nm to the autoionizing $3p^3(^2D^o)5s$; $^1D_2^o$ level. The 130.091 nm probe photons were generated by four wave difference frequency mixing (DFWM) using two 212.55 nm photons and one 580.654 nm photon in a stainless-steel cell filled with an Ar/Kr mixture (3:1 mixing ratio). The laser light at 212.55 nm was produced by doubling the output of a 355 nm (Nd:YAG laser) pumped dye laser (Sirah, PESC-G-24) operating at ~425 nm. A portion of 532 nm output of the same Nd:YAG laser was used to pump another dye laser (Sirah, PESC-G-18) which operated at ~580 nm. The polarization vector of the probe laser $\boldsymbol{\varepsilon}_{\mathbf{probe}}$ was determined by the polarization of the 580 nm radiation**,** which was fixed in the vertical direction. The resulting S^+ $(^1D_2^o)$ ions were then accelerated through the remainder of the IOA and passed through a 740 mm long field-free region before impacting on a 75 mm-diameter chevron double MCP detector coupled with a P43 phosphor screen. Transient images on the phosphor screen were recorded by a charge-coupled device (CCD) camera (Imager pro plus 2M, La Vision), using a 30 ns gate pulse voltage in order to acquire time sliced images. Images were taken under different experimental conditions to confirm that the signal was from the intended two-colour VUV pump-probe scheme. Specifically, images were recorded: (i) with both pump (VUV FEL) and probe (VUV DFWM) beams present in the interaction region; (ii) with the pump beam present but the probe beam blocked, and (iii) with the pump beam blocked and the probe beam

present. The one-color background images recorded under conditions (ii) and (iii), which were very weak compared to the image measured with both beams present, were subtracted from the two-color image recorded under condition (i). The relative timings of the gas pulse from the pulsed molecular beam, of the pump and probe laser photons in the interaction volume, and of the detector gate pulse were controlled by two delay generators (DG645, Stanford Research Systems). Converting the radius of any given feature in the measured images to the corresponding $S(^1D_2)$ atom velocity relied on calibration factors derived from imaging O^+ ions from the one colour multiphoton excitation of O_2 at λ = 225.67 nm.[9]

Supplementary References

[1] Shiell, R. C., Hu, X. K., Hu, Q. J. & Hepburn, J. W. A determination of the bond dissociation energy (D_0(H–SH)): Threshold ion-pair production spectroscopy (TIPPS) of a triatomic molecule. *J. Phys. Chem. A* **104**, 4339-4342 (2000).

[2] Linstrom, P. J. & Mallard, W. G. Eds., NIST Chemistry WebBook, NIST Standard Reference Database Number 69**,** National Institute of Standards and Technology, Gaithersburg MD, 20899, https://doi.org/10.18434/T4D303, (retrieved October 29, 2019).

[3] Zhou, W. D., Yuan, Y., Chen, S. P. & Zhang, J. S. Ultraviolet photodissociation dynamics of the SH radical. *J. Chem. Phys*. **123**, 054330 (2005).

[4] Kramida, A., Ralchenko, Yu., Reader, J. & NIST ASD Team (2019). *NIST Atomic Spectra Database* (version 5.7.1), [Online]. Available: https://physics.nist.gov/asd [Tue Oct 29 2019]. National Institute of Standards and Technology, Gaithersburg, MD. DOI: https://doi.org/10.18434/T4W30F.

[5] Komasa, J., Piszczatowski, K., Łach, G., Przybytek, M., Jeziorski, B. & Pachucki, K. Quantum electrodynamics effects in rovibrational spectra of molecular hydrogen. *J. Chem. Theor. Comput*. **7**, 3105-3115 (2011).

[6] Gorman, M. N., Yurchenko, S. N., Tennyson, J. ExoMol molecular line lists XXXVI: $X^2\Pi$–$X^2\Pi$ and $A^2\Sigma^+$–$X^2\Pi$ transitions of SH. *MNRAS* **490**, 1652-1665 (2019).

[7] Chang, Y., et al. Hydroxyl super rotors from vacuum ultraviolet photodissociation of water. *Nat. Commun*. **10**, 1250 (2019) and references therein.

[8] Lin, J. J., Zhou, J. G., Shiu, W. & Liu, K. Application of time-sliced ion velocity imaging to crossed molecular beam experiments. *Rev. Sci. Instrum*. **74**, 2495-2500 (2003).

[9] Parker, D. H. & Eppink, A. T. J. B. Photoelectron and photofragment velocity map imaging of state-selected molecular oxygen dissociation/ionization dynamics. *J. Chem. Phys*. **107**, 2357-2362 (1997).
